# Where Enhanced Recovery after Surgery (ERAS) Protocols Meet the Three Major Current Pandemics: COVID-19, Obesity and Malignancy

**DOI:** 10.3390/cancers14071660

**Published:** 2022-03-25

**Authors:** Anastasia Prodromidou, Aristotelis-Marios Koulakmanidis, Dimitrios Haidopoulos, Gregg Nelson, Alexandros Rodolakis, Nikolaos Thomakos

**Affiliations:** 1 Gynaecologic Oncology Unit, 1st Department of Obstetrics and Gynaecology, Alexandra Hospital, National and Kapodistrian University of Athens, 11528 Athens, Greece; aristoteliskoulak@gmail.com (A.-M.K.); dimitrioshaidopoulos@gmail.com (D.H.); a.rodolaki@gmail.com (A.R.); thomakir@hotmail.com (N.T.); 2 Department of Obstetrics and Gynecology, University of Calgary, Calgary, AB T2N 1N4, Canada; gregg.nelson@albertahealthservices.ca

**Keywords:** COVID-19, gynecologic oncology, obesity, SARS-CoV-2, malignancy, cancer

## Abstract

**Simple Summary:**

The SARS-CoV-2 (COVID-19) pandemic has significantly modified the medical services provided for patients that receive care either for COVID-19 or for those that need care for benign diseases, including obesity, or for malignant ones, such as gynecological cancer. We sought to investigate the association among three major worldwide health issues (COVID-19, obesity, and malignancy) and how ERAS protocols can potentially provide optimal management of patients with obesity and malignancy during the COVID-19 pandemic, with special attention to patients who required surgery for gynecologic oncology. We strongly believe that the application of ERAS protocols could play a key role during these unprecedented COVID-19 times.

**Abstract:**

The outbreak of the SARS-CoV-2 (COVID-19) pandemic has transformed the provision of medical services for both patients that receive care for COVID-19 and for those that need care either for benign diseases, including obesity, or for malignancies, such as gynecological cancer. In this perspective article, we focus on the association among three major worldwide health issues and how ERAS protocols can potentially provide optimal management of patients with obesity and malignancy during the COVID-19 pandemic, with special attention to patients who required surgery for gynecologic oncology. A thorough search of the literature on the respective topics was performed. Patients with malignancy and obesity presented with increased vulnerability to COVID-19 infection. However, the management of their disease should not be withheld. Protective measures should be established to reduce exposure of patients with oncological diseases to SARS-CoV-2 while simultaneously enabling their access to vaccination. Since ERAS protocols have proved to be efficient in many surgical fields, including gynecologic oncology, general surgery, and orthopedics, we strongly believe that ERAS protocols may play a significant role in this effort. The end of the COVID-19 pandemic cannot be accurately predicted. Nevertheless, we have to ensure the appropriate and efficient management of certain groups of patients.

## 1. Introduction

The outbreak of SARS-CoV-2 (COVID-19) and its subsequent declaration by the World Health Organization (WHO) as a pandemic on 11th March 2020 has transformed the provision of medical services for both patients that receive care for COVID-19 and those that need care for other benign or malignant diseases. The pandemic has had significant professional and psychological consequences for healthcare providers. During the year 2021, the introduction of vaccines has brought significant hope for immunity against the disease for society as a whole; however it is still markedly uneven due to the inequity of vaccine access and the emergence of novel viral variants [1]. Furthermore, the different immune susceptibilities of the new variants has raised concerns regarding the amount of viral load in the community and the expansion of disease transmission [1].

Pandemics and cancer present similarities in growth and risk models and are both leading causes of mortality worldwide [2]. Despite the significant advances in modern therapies and quality of treatment, malignant diseases are among the most fatal conditions globally. A rise of approximately 50% in cancer cases is expected in 2040 compared with in 2020 [3]. According to the WHO, cancer is the second leading cause of death worldwide, with an estimate of 9.6 million cancer-related deaths in 2018; that can be translated to one in six deaths [3,4]. Cancer exerts significant psychological, physical, and economic burdens on individuals, societies, and healthcare systems. Gynecologic malignancies, including mainly cervical, endometrial, and ovarian cancer, are associated with significant morbidity and mortality among the gynecologic population [5]. A variety of clinicopathological characteristics have shown effects on the prognosis of gynecologic oncology patients [5]. Obesity not only is considered a risk factor for the development of certain types of gynecologic malignancies but also has been associated with poorer surgical outcomes [6].

Obesity has reached pandemic proportions worldwide, with estimated overweight and obese populations of approximately 39% and 13%, respectively [7]. Obesity is a major global health concern because of its overwhelming effect on an individual’s health, which correlates with high rates of morbidity, including elevated risk of infection, respiratory and cardiometabolic diseases, as well as the development of malignancy [7]. Furthermore, the socioeconomic impact of obesity constitutes a huge burden, related not only to an excess of healthcare expenditure but also to critical loss of public productivity as a result of increased mortality and permanent disability [8]. Especially during the COVID-19 pandemic, patients with obesity suffered from a number of penalties that affected both populations with and without infections [9]. In particular, patients with obesity and a COVID-19 infection have a worse prognosis, which is mainly related to their comorbidities and impaired immune system [9]. Furthermore, patients with obesity require special care by qualified medical staff and using equipment that are not always available [9]. Patients without COVID-19 infections, on the other hand, are isolated at home during the pandemic; have limited access to potential planned surgical procedures; and are restricted in their choice of physical activity, which can worsen their already fragile medical condition [9].

During recent decades, Enhanced Recovery After Surgery (ERAS) protocols have been applied in a variety of surgical subspecialties. They were initially proposed for patients who underwent surgery for colorectal cancer and aimed to hasten postoperative recovery while simultaneously decreasing postoperative morbidity and readmissions [10,11]. By introducing a variety of standardized pre-, intra-, and postoperative modalities, the ERAS protocols have shown expedited functional recovery through attenuation of the stress response [11].

In this perspective article, we focus on the association among three major worldwide health issues and how ERAS protocols can potentially provide optimal management of patients with obesity and malignancy during the COVID-19 pandemic. We specifically focus on patients with gynecologic malignancies who underwent surgery, which represents a specific population of patients with cancer.

## 2. COVID-19 and Malignancy

The ongoing COVID-19 pandemic has dramatically changed the characteristics of medical care for patients with malignant diseases. A variety of unknown and difficult-to-solve problems have arisen, especially in daily surgical clinical practice. After an interval of about 6–8 months of almost total cancellation of all elective surgeries, the surgical community was called upon to take effective measures in preventing in-hospital SARS-CoV-2 spread so as to resume elective surgical procedures with the greatest safety for the patient [12]. Patients with oncological diseases have faced significant delays in their cancer diagnoses and treatments during the COVID-19 pandemic era. In particular, the diagnostic workflow of patients suspected of having cancer has been withheld due to the limited access of those patients to healthcare services and diagnostic procedures during the COVID-19 pandemic. Consequently, this has resulted in significant delays in cancer diagnosis, which advanced the stage of disease at diagnosis and the number of potentially avoidable cancer-related deaths [13]. This is also reflected in the decreased number of new cancer diagnoses during the COVID-19 pandemic due to the restrictions and alterations in health-seeking guidelines [13]. Decisions about the management of patients with oncological diseases should balance the need for proceeding with cancer treatment with the reported elevated susceptibility to COVID-19 infection and with the subsequent potentially poor outcomes of patients with COVID-19 infections and cancer [14]. In order to proceed with the surgical management of patients with oncological diseases, a plethora of preventative measures have been proposed by several surgical societies. Among them, restrictions regarding hospital visits unless absolutely necessary, limitations in the number of family members accompanying the patient, pre-operative SARS-CoV-2 screening before admission to the hospital and frequently thereafter during hospital stay, isolation of patients for a couple of days before surgery, and attempts to reduce hospital stays after surgery have been proposed. Some ongoing studies aim to elucidate the exact role of preoperative SARS-CoV-2 testing and remote prehabilitation in patients who are scheduled for elective surgeries [15]. In any case, all appropriate measures should be taken to ensure the availability of an operating theatre, surgeons, hospital staff, and resources in order for the cancer surgery to be prioritized [16]. Consequently, there is a need to establish perioperative pathways to hasten recovery and to increase hospital capacity [16]. ERAS protocols could serve as a major tool in helping combat this problem [16].

Furthermore, special attention should be paid to the characteristics and care of patients with cancer who are diagnosed with SARS-CoV-2. According to some studies, patients with malignancies are at higher risk of developing a COVID-19 infection [17]. However, no firm conclusion can be derived based on the current literature regarding the exact interaction between SARS-CoV-2 and cancer since there are many cofounders that can influence the course of those patients including age, comorbidities, smoking, and obesity. The potential suppression of the immune system of patients with cancer who undergo anti-cancer therapy explains the vulnerability of this group of patients with malignant diseases [17,18]. However, this could not be the case for all patients with cancer. Furthermore, cancer-related hypercoagulopathy could further increase the morbidity of these patients [18]. Therefore, prevention, early recognition, and appropriate management of thrombosis cases could be an important tool in reducing patients’ morbidity.

We identified three studies in the literature that compared the differences in characteristics and outcomes among 5542 COVID-19 infected patients with (*n* = 398) and without malignancies (*n* = 5144) [19,20,21]. Their outcomes are summarized in Table 1.

The presence of comorbidities was more prevalent in patients with cancer who were infected with SARS-CoV-2. As shown in Table 1, mortality rates were controversial among the included studies. The multivariate analysis performed by Dai et al. revealed that the elevated risk of mortality, the presence of severe symptoms, ICU admission, and mechanical ventilation remained significant for patients with cancer who were infected by COVID-19 [19]. The same authors performed a separate analysis among patients with metastatic and non-metastatic cancer and proved that, concerning the aforementioned parameters, significance was only retained for patients with metastasis [19]. Additionally, patients who received surgery and immunotherapy presented with elevated mortality and increased incidence of severe symptoms, while this was not observed for those under radiotherapy [19]. Finally, Aboueshia et al. detected no difference in mortality rates among patients with cancer who were currently under treatment (active) and those who were not (non-active) [21].

The management of patients with malignancy during the COVID-19 pandemic is of critical importance. However, the available guidelines by existing committees are not yet clear on the optimal approach regarding patients with malignancy who were or were not infected with COVID-19 during the pandemic. Further trials and audits from high-volume centers are warranted to elucidate whether COVID-19 infection and malignancy correlate with higher mortality, and to identify potential biomarkers used to stratify the risk of mortality and development of severe complications in those patients. The establishment of strategies and modalities to protect patients with cancer from SARS-CoV-2 infection during their treatment and to adjust their management against cancer during a COVID-19 episode would be beneficial for this evidently high-risk group of patients.

## 3. COVID-19 and Obesity

The restrictions to physical activity and the potentially unhealthy eating habits that have been adopted during the COVID-19 pandemic could be considered as additional risk factors that predispose a person to obesity. Similar to other infectious diseases, a COVID-19 infection has been claimed to induce obesity. The potential mechanisms that have been proposed include the increase in adipogenesis and in chronic inflammation that promote fatty tissue angiogenesis [22]. Finally, the pandemic has paused elective bariatric procedures, and thus, the management of obesity in patients was withheld, leading to a significant expansion of the adverse consequences of obesity including cardiovascular complications, diabetes mellitus, and cancer [22]. However, the outcomes from a single high-volume center in Canada showed that the application of ERAS protocols kept the bariatric program fully functional during the pandemic, allowing for discharges on the first postoperative day [23].

As mentioned above, patients with obesity can have compromised immune systems with a low-grade inflammatory state as well as respiratory dysfunction, indicating a potential relationship between obesity and the severity of SARS-CoV-2 disease. A recent meta-analysis by Cai et al. showed that patients with obesity and SARS-CoV-2 were more likely to be hospitalized, to suffer from more severe disease, to be admitted to the ICU, and to receive mechanical ventilation more often compared with patients without obesity [7]. The mortality rates of those patients were accordingly elevated [7]. Susceptibility to acute respiratory distress syndrome (ARDS), which constitutes the primary cause of mortality due to SARS-CoV-2, is considerably greater among patients with obesity. There is strong evidence suggesting that a higher body mass index (BMI) is greatly associated with COVID-19 infection, with an estimated risk increase of about 5–10% of hospitalization due to SARS-CoV-2 for every kg/m^2^ excess of BMI [24]. In addition, patients with obesity are at a higher risk for reduced effectiveness of COVID-19 vaccination, which can be potentially attributed to metabolic dysfunction, leading to a weakened immune response [25].

## 4. The Triangle of Pandemic Doom

**Figure 1:**
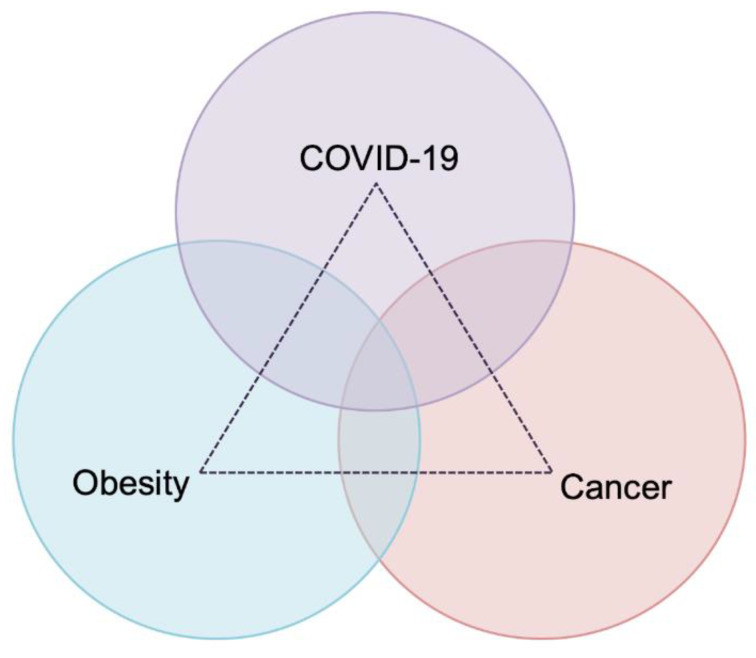
The triangle of pandemic doom.

As previously highlighted, appropriate clinical decision-making for patients undergoing surgery during these unprecedented times is of paramount importance in order to achieve optimal outcomes, while a dangerous triangle of doom is forming (Figure 1). Patients with obesity undergoing surgery for cancer and imperiled by COVID-19 infection find themselves at a very high risk for perioperative complications and mortality. At this point, the implementation of ERAS protocols may serve as a life jacket for patients who find themselves within this deleterious triangle.

## 5. ERAS and Surgical Oncology

The application of ERAS fast-track protocols has been proposed as a tool for improving the perioperative care of patients and aiming to decrease postoperative morbidity, hospital stay, and hospitalization costs. The main goal of applying ERAS protocols is to hasten the return of patients to normal activity. It is known that surgical operations and hospital stays can interfere with normal homeostasis, a phenomenon that is called the surgical stress response (SSR) and involves the immune and neuroendocrine systems [26]. The decrease in SSR could lead to an optimal postoperative course with significant reduction in postoperative morbidity [26]. In that context, the application of ERAS protocols could contribute to protection against SSR. This can be achieved by encompassing strategies to eliminate perioperative opioid use and to introduce early oral food intake and ambulation, as well as prudent fluid administration. The components of ERAS protocols are classified into pre-, intra-, and postoperative components [27]. There is a significant interaction among the ERAS components, with one affecting the other [28]. Some of the main components of ERAS protocols in gynecologic oncology are shown in Table 2.

The cooperation of a multidisciplinary team consisting of surgeons, anesthesiologists, nutrition specialists, nursing staff, and physiotherapists is of critical importance to achieve the optimal postoperative care [28]. To that end, the proper education of all these specialties could lead to the successful application of ERAS protocols. A plethora of original studies and reviews have demonstrated the superiority of ERAS protocols in many surgical fields in ameliorating short-term outcomes including a significant reduction in complication rates and hospital stays with no impact in reoperation and readmission rates [29,30]. However, less is known about the long-term efficacy of ERAS protocols in patients with malignancy. According to the findings by Gustafsson et al., the application of ERAS protocols in patients with colorectal cancer undergoing surgery was shown to be associated with improved 5 year disease specific survival [31]. Interestingly, the maintenance of fluid balance, the prevention of fluid overload, and monitoring of calories by oral intake at the day of surgery were considered the ERAS components that were independently related to improved 5 year survival outcomes [31]. ERAS can be applied in all patients who have been selected to receive surgical management for their disease.

### 5.1. ERAS and Gastrointestinal Surgery

The use of ERAS-based clinical pathways for patients who had pancreatoduodenectomy due to pancreatic cancer has been shown to be effective for both increased patient care and reduced hospital costs according to the meta-analysis by Karunakaran et al. [32]. The authors recorded a significant decrease in hospital stays, complications, and overall hospital costs through the ERAS arm of care compared with standard care [32]. The respective benefits have also been seen in patients with gastric cancer who underwent surgery with preoperative education, early rehabilitation with mobilization, and first postoperative day oral feeding [33]. In liver surgery, the application of ERAS resulted in a significant reduction in complications and length of stay with no impact to mortality and re-admission rates [34]. Furthermore, for patients with colorectal cancer who had laparoscopic surgery, the application of ERAS was associated with shorter hospital stays, and earlier time to first flatus and defecation, based on the outcomes of a meta-analysis of 13 randomized clinical trials [35].

### 5.2. ERAS and Gynecologic Oncology Surgery

ERAS protocols have also been used in patients with gynecologic cancers. According to a recent meta-analysis by Bisch et al., the application of ERAS protocols in patients with gynecologic malignancy has been associated with significant benefits for the patients’ postoperative course by reducing the length of hospital stays and postoperative complications with no impact in readmission rates and mortality [36]. Additionally, Tankou et al. compared the postoperative outcomes of patients with advanced ovarian cancer who had interval debulking surgery after the application of neoadjuvant chemotherapy before and after ERAS [10]. They showed a significantly elevated proportion of patients that resumed chemotherapy at 28 days after surgery in the post-ERAS group compared with those in the pre-ERAS group (80% vs. 64%, odds ratios 2.29, *p* = 0.002) [10]. The ERAS Society has issued and updated guidelines on the optimal perioperative care of patients with gynecologic malignancy that aimed to improve patients’ postoperative outcomes [37].

### 5.3. ERAS and Urological Surgery

The use of ERAS has also been extensively investigated in patients who had surgery due to urological indications. More specifically, for patients with bladder cancer who had radical cystectomy, those who were managed under ERAS protocols had a shorter time to first bowel movement and a shorter hospital stay compared with the group without ERAS management. No difference was observed in the 30-day readmission and complication rates [38]. ERAS protocols were also shown to be beneficial in terms of time to first flatus, increasing safety in catheter removal and reducing hospital stay in patients who had radical prostatectomy [39].

### 5.4. ERAS and Head and Neck Surgery

The current literature also presents encouraging perioperative outcomes in the use of ERAS in patients with head and neck cancers. In particular, there is growing evidence for the clinical and financial benefits of ERAS in major head and neck surgery [40]. Early oral intake and trachea-stoma closure have been recorded as the key beneficial components of ERAS in these surgeries [41]. However, data are still limited in the field and further, larger, well-designed trials are required to validate the safety and feasibility of ERAS protocols in head and neck surgery [41,42].

## 6. Key Role of ERAS in Gynecologic Oncology

Patients with malignant diseases are more susceptible to SARS-CoV-2 due to cancer-related immunosuppression. Therefore, there is an urgent need to develop strategies to reduce exposure to COVID-19 in patients with cancer in need of surgical intervention. ERAS protocols have been proposed as valuable tools in the surgical management of patients with malignancy during the SARS-CoV-2 era [16]. These protocols maintain homeostasis during the perioperative period, aiming to minimize the prevalence and severity of complications after complex gynecologic oncology surgeries even during the COVID-19 pandemic [12]. More specifically, strategies for the reduction in the length of hospital stays and readmissions are among the preventative measures of transmission of SARS-CoV-2 [43]. Moreover, a shorter length of hospitalization can improve the mental well-being not only of patients after surgery but also of their care providers and relatives, who are restricted from hospital visits, and can thus result in more favorable postoperative outcomes [43]. Finally, the implementation of ERAS protocols also seems to be cost-effective: the increase in total savings per cancer patient allows for the opportunity to redistribute these savings to other areas of the healthcare system. It is obvious that the combination of obesity and malignancy expands the risks of suffering from SARS-CoV-2 and the severe complications of the disease. During this unknown and difficult period, there is an increased need for the development of perioperative care pathways that will ensure the safety of patients with obesity and gynecologic malignancies.

The use of ERAS protocols in gynecologic oncology has shown reduced lengths of hospital stays, which can also minimize the risk of COVID-19 infection. Additionally, in patients with obesity and gynecologic malignancies, the use of ERAS protocols has been proven to be safe and efficient, with comparable perioperative outcomes to patients without obesity [44]. The implementation of ERAS protocols in patients who required surgery for gynecologic oncology and who had minimally invasive hysterectomy was associated with significantly increased same-day discharge rates: 75% following ERAS protocols as compared with 29% during the pre-implementation era, with no impact in complication and readmission rates [45]. Interestingly, the mean BMI of the study population was 32, with no observable difference in BMI among the pre-ERAS and post-ERAS intervention groups [45]. This clearly indicates the applicability of the suggested, minimally invasive ERAS program in patients with obesity that can also facilitate better hospital management of patients with obesity who require surgery for malignancy during the COVID-19 pandemic. In addition, ERAS protocols promote early functional recovery after surgery, resulting in lower rates of complications and a faster return to the intended oncology treatment, compared with traditional methods in patients with a high risk of postoperative morbidity, such as those with obesity. Therefore, the application of the main components of ERAS, such as those mentioned in Table 2, with further special consideration to some specific elements, can contribute to the optimal management of patients with obesity, COVID-19 infections, and oncologic diseases. Moreover, emphasis should be given to prehabilitation strategies that promote weight loss and exercise; the application of a preoperative low-calorie diet and improvements in general fitness and respiratory capacity are of paramount importance. Postoperative dietary and nutritional support is equally important in providing early nutritional care to patients with oncological diseases during the immediate postoperative period. As for the anesthesiology part, the anesthesiologist should be aware of the challenges of intubation of patients with obesity and adopt lung protective strategies with adjustment in ventilation parameters and positioning that can improve gas exchange and pulmonary mechanisms. ERAS protocols enable safe and effective treatment options for patients with obesity, while human and institutional resources are preserved for patients with SARS-CoV-2 requiring hospitalization. Women with obesity represent a significant proportion of patients with gynecologic malignancies and the postponement or the cancellation of their management will lead to a further peak of cancer-related deaths added to those due to COVID-19 infection.

## 7. Conclusions

No one can accurately predict when the COVID-19 pandemic will end. However, we have to ensure the appropriate and efficient management of patients with oncological diseases during these unprecedented times. Greater attention should be paid to patients with obesity, which constitute a high-risk group of patients. Consequently, there is a strong need to establish strategies to eliminate the adverse outcomes that can arise from the combination of malignancy, obesity, and COVID-19 infection. Despite the increased vulnerability of patients with obesity and cancer to COVID-19 infection, the management of their disease should not be withheld. To that end, we should ensure that management strategies consist of protective measures to reduce their exposure to SARS-CoV-2, and unfettered access to vaccination. Since ERAS fast-track protocols have been proven to be effective in gynecologic oncology surgery and other surgical oncology disciplines, we strongly believe that ERAS fast-track protocols may play a significant role in efforts to combat the serious “triangle of pandemic doom”.

## Figures and Tables

**Table 1:** Studies reporting characteristics and outcomes of patients with COVID-19 with malignancy versus without malignancy.

Year; Author	2021; Aboueshia	2021; Mohamed	2020; Dai
**Country**	USA, Egypt	USA	China, USA
**Type of study**	RS	RS	MS-PS
**Study period**	February 2017–April 2020	March 2020–April 2020	January 2020–February 2020
**Inclusion criteria**	Adult patients hospitalized with COVID-19	Patients who are positive for COVID-19 who had testing due to fever or signs/symptoms suggestive of respiratory illness, history of travel to affected areas, direct contact with a person who was confirmed as having a COVID-19 infection	Patients with or without cancer who were infected with COVID-19 matched by age
**Evaluated outcomes**	Relationship between cancer and severe COVID-19 illness with adverse outcomes/in-hospital mortality, ICU admission, risk of intubation, duration of mechanical ventilation, LOS	Difference between patients with COVID-19 and with and without cancer in demographics, clinical and behavioral characteristics; prediction of mortality in patients with cancer	Death; ICU admission; severe clinical symptoms; acute kidney injury; disseminated intravascular coagulation; rhabdomyolysis
**Patient No**	57 vs. 203	236 vs. 4405	105 vs. 536
**Age (years)**	63.6 ± 12.5 ^a^ vs. 58.7 ± 14.6 ^a^ *p* = 0.023	69 (61–78) vs. 57 (40–70) *p* < 0.001	64 (14) ^b^ vs. 63.5(14) ^b^ *p* = 0.25
**Most common type of cancer**	Breast and prostate	N/A	Lung cancer
**ICU admission (%)**	22.2% vs. 16.1% *p* = 0.07	N/A	OR 2.84 95% CI 1.59–5.08 *p* < 0.01
**Complications (%)**	78.8% vs. 79.9% *p* = 0.84	N/A	N/A
**Mechanical ventilation N (%)**	12 (26.1%) vs. 52 (32.9%) *p* = 0.47 (closed cases)	N/A	11(10.48%) vs. 47 (8.77%) *p* = 0.58 (non-invasive) 11(10.48%) vs. 15(2.79%) *p* < 0.001
**Mortality (%)**	12.3% vs. 16.3% *p* = 0.53	29 (12.3%) vs. 357 (8.1%) *p* = 0.023	OR 2.34 95% CI 1.15–4.77 *p* = 0.03
**Discharged patients N (%)**	42/49 (85.7%) vs. 142/175 (81.1%)	75 (31.8%) vs. 2026 (46%) *p* < 0.001	N/A
**LOS**	12.8 ± 11.4 ^a^ vs. 8.58 ± 6.5 ^a^ *p* = 0.002	N/A	27.01 ± 9.52 vs. 17.75 ± 8.64 *p* < 0.01

RS: Retrospective; MS: multicenter; ICU: intensive care unit; LOS: length of stay, ^a^ mean ± SD, ^b^ median (IQR).

**Table 2:** Key principles of ERAS protocols in gynecologic /oncology.

Thorough preoperative counseling.
Preoperative prehabilitation and optimization (cessation of smoking and alcohol abuse, and correction of possible anemia).
No mechanical bowel preparation.
Clear fluids consumption (oral carbohydrate drinks): until 2 h preoperatively and a light meal 6 h prior to the introduction of anesthesia.
No administration of preoperative sedatives for anxiety reduction.
For surgery > 30 min, dual VTE prophylaxis administration: including mechanical and either LMWH or heparin.
Administration of first-generation cephalosporins and anaerobic prophylaxis (in case of bowel resection) 60 min prior to incision.
Short-acting anesthetics and local anesthesia wound infiltration.
Use > 2 antiemetic agents for PONV prevention.
No routine use of nasogastric intubation. If inserted during surgery, remove immediately after surgery.
No use of surgical drains.
Preservation of normothermia and euvolemia intra-operatively.
Early discontinuation of intravenous fluids postoperatively (once tolerating oral fluids) and simultaneous return to regular diet within the first 24 h postoperatively.
Maintenance of blood glucose levels < 180–200 mg/dL, and if glucose levels surpass this range, use insulin infusions.
Opioid sparing strategies with multimodal analgesia.
Remove bladder catheter at <24 h postoperatively.
Active mobilization from the first postoperative day.

VTE: venous thromboembolism; LMWH: low molecular weight heparin; PONV: postoperative nausea and vomiting.
